# Influence of Hypoxia on Radiosensitization of Cancer Cells by 5-Bromo-2′-deoxyuridine

**DOI:** 10.3390/ijms23031429

**Published:** 2022-01-27

**Authors:** Magdalena Zdrowowicz, Paulina Spisz, Aleksandra Hać, Anna Herman-Antosiewicz, Janusz Rak

**Affiliations:** 1 Laboratory of Biological Sensitizers, Faculty of Chemistry, University of Gdańsk, 80-308 Gdańsk, Poland; magdalena.zdrowowicz@ug.edu.pl (M.Z.); paulina.spisz@ug.edu.pl (P.S.); 2 Department of Medical Biology and Genetics, Faculty of Biology, University of Gdansk, 80-308 Gdańsk, Poland; aleksandra.hac@ug.edu.pl (A.H.); anna.herman-antosiewicz@ug.edu.pl (A.H.-A.)

**Keywords:** radiosensitizer, radiotherapy, X-ray, modified nucleosides, DNA damage, hypoxia

## Abstract

Radiotherapy is a crucial cancer treatment, but its outcome is still far from satisfactory. One of the reasons that cancer cells show resistance to ionizing radiation is hypoxia, defined as a low level of oxygenation, which is typical for solid tumors. In the hypoxic environment, cancer cells are 2–3 times more resistant to ionizing radiation than normoxic cells. To overcome this important impediment, radiosensitizers should be introduced to cancer therapy. When modified with an electrophilic substituent, nucleosides may undergo efficient dissociative electron attachment (DEA) that leaves behind nucleoside radicals, which, in secondary reactions, are able to induce DNA damage, leading to cancer cell death. We report the radiosensitizing effect of one of the best-known DEA-type radiosensitizers—5-bromo-2′-deoxyuridine (BrdU)—on breast (MCF-7) and prostate (PC3) cancer cells under both normoxia and hypoxia. MCF-7 and PC3 cells were treated with BrdU to investigate the effect of hypoxia on cell proliferation, incorporation into DNA and radiosensitivity. While the oxygen concentration did not significantly affect the efficiency of BrdU incorporation into DNA or the proliferation of tumor cells, the radiosensitizing effect of BrdU on hypoxic cells was more evident than on normoxic cells. Further mechanistic studies performed with the use of flow cytometry showed that under hypoxia, BrdU increased the level of histone H2A.X phosphorylation after X-ray exposure to a greater extent than under normal oxygenation conditions. These results confirm that the formation of double-strand breaks in hypoxic BrdU-treated cancer cells is more efficient. In addition, by performing stationary radiolysis of BrdU solution in the presence of an ^●^OH radical scavenger, we compared the degree of its electron-induced degradation under aerobic and anaerobic conditions. It was determined that radiodegradation under anaerobic conditions was almost twice as high as that under aerobic conditions.

## 1. Introduction

Radiotherapy (RT) continues to be a mainstay in the treatment of a variety of cancers and is used in more than 50% of patients, both for curative and for palliative purposes [1]. Unfortunately, the therapeutic effects of ionizing radiation (IR) are reduced by radioresistance factors [2]. Hypoxia—a common feature of solid tumors that results from an imbalance between oxygen availability and consumption—is defined as one of the most important reasons for radiotherapy failure [3]. Indeed, since the 1950s [4], it has been repeatedly demonstrated that molecular oxygen significantly modifies the effectiveness of radiotherapy [5,6]. The response of the anoxic regions of the tumor to radiotherapy is 2.5–3 times weaker relative to that of well-oxygenated cells. This effect is known as the oxygen enhancement ratio, and it is most commonly explained by the oxygen fixation hypothesis [7,8]. This radiochemical rationale postulates that radical-induced DNA damage can be “fixed” (made permanent and irreparable) by molecular oxygen, rendering DNA damage irreparable. Indeed, if oxygen is available, it can react with DNA damaged by IR (DNA^•^), creating stable organic peroxides (DNA-O-O^•^), which are not readily repaired by the cell. In contrast, the damaged biopolymer (DNA-H) is easily repaired in the absence of oxygen, restoring it to the original state [7,9].

One of the ways to overcome radioresistance is by introducing radiosensitizers to the radiation treatment [10]. Radiosensitizers are chemicals or pharmaceuticals that can enhance cancer cell sensitivity to IR by accelerating DNA damage and producing free radicals indirectly [11]. These substances were initially classified into five groups based on the mechanisms of their action: (1) suppressors of endogenous radioprotective substances, (2) cytotoxic substances formed by radiolysis of the radiosensitizer, (3) inhibitors of DNA repair, (4) thymine analogs that can incorporate into DNA and (5) oxygen mimetics that can imitate the action of oxygen [12]. However, the continuous development and innovation of new technologies and strategies of radiosensitization forced the introduction of a new classification into three categories based on sensitizer structures: (1) small molecules, (2) macromolecules and (3) nanomaterials [10].

Modified nucleosides (MN), which are small-molecule radiosensitizers, are geared towards hydrated electrons [13]. In the absence of oxygen, electrons, besides hydroxyl radicals, are the most abundant products of water radiolysis. The former undergo very fast thermalization and become hydrated (e_hyd_) within a picosecond time scale. Interest in the role of electrons in the context of generating DNA damage and radiosensitization increased when Sanche and coworkers demonstrated the efficient generation of single- and double-strand breaks (SSBs and DSBs) in plasmid DNA under ultrahigh vacuum by electrons with energies below the ionization threshold of DNA [14]. Although native DNA binds solvated electrons in aqueous solutions [15], strand breaks do not occur [16,17], probably due to swift protonation of the resulting anions [18]. In order to counteract this unfavorable (from a radiotherapy viewpoint) situation, one can use substances that “activate” solvated electrons in the context of DNA damage. Such substances can be modified nucleosides, which utilize electrons for their radiosensitizing activity [19]. Electron attachment to electrophilic nucleosides causes efficient dissociative electron attachment (DEA), leading to reactive radicals [20,21]. If such a modified nucleobase is incorporated into the DNA molecule, then the reactive radical, a product of DEA, may enter secondary chemical reactions that lead to a strand break or other type of DNA damage. One of the most thoroughly studied radiosensitizers of this type is 5-bromo-2′-deoxyuridine (BrdU). This is a highly electron-affinic molecule and is susceptible to DEA that leads to the reactive uracil-5-yl radical via the unstable BrdU anion. This derivative is a good substrate for human thymidine kinase and DNA polymerase [22], and thus, BrdU is easily transformed into 5′-triphosphate of 5-bromo-2′-deoxyuridine (BrdUTP) and then effectively incorporated into cellular DNA [23]. It has been demonstrated that the radiolysis of deoxygenated aqueous solutions containing labeled oligonucleotides produces a significant number of single-strand breaks (SSBs). Studies employing native and brominated trinucleotides, TXT (where X = U/BrU, C/BrC, A/BrA or G/BrG), and radical scavengers have offered proof that only the labeled oligonucleotides undergo damage, mainly in the form of SSBs. It was also demonstrated that the presence of BrdU in a DNA strand led to a three-fold increase in the susceptibility of cells to death by high-energy radiation. Despite the fact that BrdU has been the subject of both in vitro [24,25] and in vivo [26,27] experiments for many decades, comprehensive and direct studies showing the effect of hypoxia on the radiosensitizing properties of BrdU are still lacking. In this paper, we show the influence of the oxygenation level on the most important factors and processes determining the sensitizing properties of BrdU. Using both chemical and biological methods, cell proliferation, incorporation of BrdU into DNA and the radiosensitivity of prostate and breast cancer cells were compared under hypoxic and normoxic conditions.

## 2. Results and Discussion

### 2.1. Radiolysis

**Figure 1:**
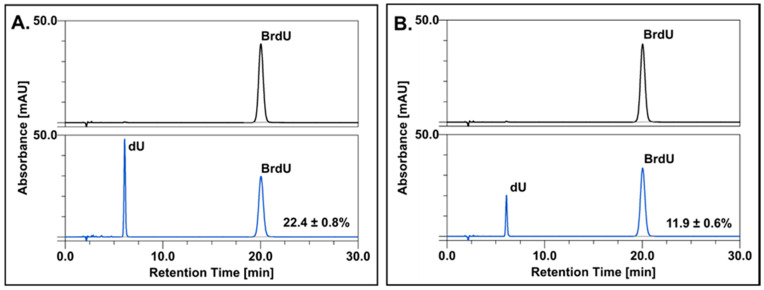
HPLC traces for a solution of BrdU before (black chromatogram) and after irradiation with a dose of 140 Gy (blue chromatogram) under anaerobic (**A**) and aerobic (**B**) conditions.

The formation of the uracil-5-yl radical as a result of DEA plays a crucial role in the radiosensitization mechanism of modified uracils [28]. In order to determine the influence of oxygenation on electron-induced degradation of BrdU, stationary radiolysis coupled with chromatographic analysis was performed. The studied solutions of BrdU were irradiated in the presence of *tert*-butanol to scavenge hydroxyl radicals. The chromatograms corresponding to the above-mentioned experiment, which was performed in anaerobic and aerobic conditions, are presented in Figure 1A,B, respectively. As expected, the final stable product of electron attachment to BrdU was 2′-deoxyuridine (dU), whose identity was confirmed by LC–MS analysis (Figure S1 in Supplementary Material). The yields of the electron-induced decomposition of BrdU were compared for both studied systems. These quantities were calculated based on the peak area of the substrate (BrdU) in the irradiated and non-irradiated solutions of the same initial concentration of BrdU. The degradation yield of BrdU under anaerobic conditions is almost twice as large compared to the normal oxygen concentration. This experiment shows unequivocally that under oxygenated conditions, solvated electrons are effectively scavenged by molecular oxygen. Since hydrated electrons have much more hampered access to the BrdU incorporated into cellular DNA than to BrdU dissolved in water, DEA to BrdU-labeled cellular DNA under aerobic conditions is probably negligible.

### 2.2. Incorporation of BrdU into Genomic DNA

DNA labeled with electron-affinic modified nucleosides that are prone to dissociative electron attachment should be, in contrast to unmodified DNA, sensitive to solvated electrons [21]. Nucleobase radicals formed within the DEA process are able to trigger serious DNA damage (strand breaks, intra- and interstrand cross-links), leading to lethal effects. In order to assess the usefulness of the studied MN, the efficiency of its incorporation into genomic DNA should be determined. Indeed, the level of radiosensitization correlates with the extent to which the analog is incorporated into DNA [29,30]. Therefore, the incorporation of BrdU into the genomic DNA of breast and prostate cancer cells was assessed in hypoxic and normoxic conditions since one could hypothesize that the oxygen status of the cell influences the efficiency of radiosensitizer incorporation into genomic DNA.

MCF-7 and PC3 cells were treated for 24 or 48 h with BrdU at a concentration of 1 µM or 10 µM. Purified DNA was enzymatically digested and analyzed by HPLC (Figure S2 in Supplementary Materials). The results in Table 1 show that the extent of labeling is dependent on the incubation time, analog concentration and cell line, but the oxygenation level does not significantly affect the studied process. The highest level of incorporation was found in the case of 48 h incubation with 10 µM BrdU, which was equal to 24.3%/23.7% (hypoxia/normoxia) for the MCF-7 cell line and was equal to 23.2%/24.2% (hypoxia/normoxia) for PC3 cells.

### 2.3. Viability of Cells Labeled with BrdU

**Figure 2:**
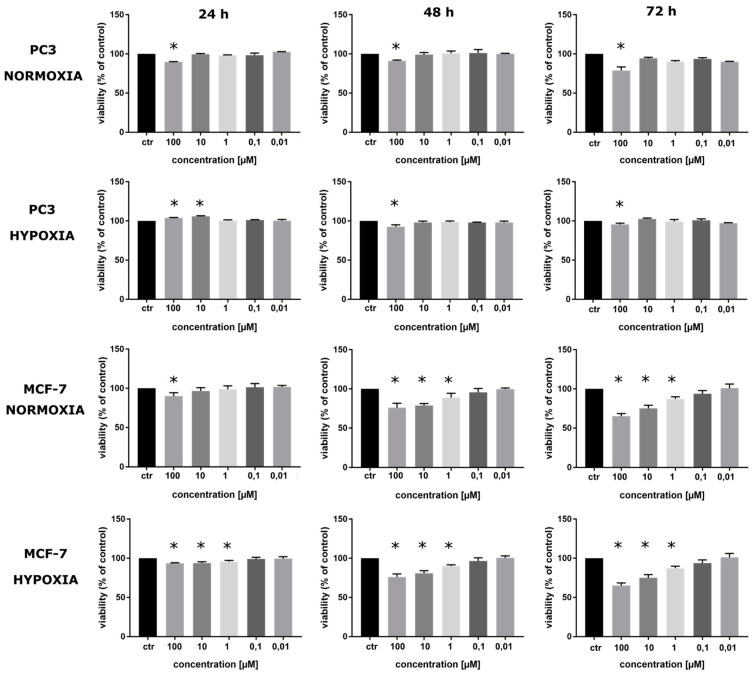
The viability of MCF-7 and PC3 cells after 24, 48 and 72 h treatment with BrdU in a range of concentrations from 0 to 100 µM under hypoxic and normoxic conditions. Results are shown as mean ± standard deviation of three independent experiments performed in triplicate. The statistical significance of the difference between treated culture compared with control (untreated culture) was determined by one-way ANOVA followed by Tukey’s multiple comparison test: * *p* < 0.05.

To determine the influence of hypoxic conditions on the cellular cytotoxicity of the studied sensitizer, the MTT test was performed. The assay was conducted for two cancer cell lines—PC3 and MCF-7—under normoxic and hypoxic conditions. The cytotoxicity of BrdU was examined at six concentrations: 0 (for the control) 100, 10, 1, 0.1 and 0.01 µM. The obtained results (Figure S3 in Supplementary Materials) show that the highest reduction in viability is observed in cells incubated for 72 h with 100 µM BrdU. For the PC3 cell line, the absorbance level measured at 570 nm wavelength was equal to 0.85 ± 0.045 for normoxic conditions and 0.86 ± 0.015 for hypoxic conditions compared to the control (1.08 ± 0.025 and 0.90 ± 0.011, respectively). For the MCF-7 cell line, a similar dependence was observed. The decrease in absorbance for the highest concentration after 72 h incubation was equal to 1.25 ± 0.050 for the normoxic conditions and 1.13 ± 0.034 for the hypoxic ones compared to the control (1.69 ± 0.052 and 1.74 ± 0.028, respectively). Thus, the viability, expressed as % of control (Figure 2), was reduced to 78.9 ± 4.5% and 95.5 ± 1.5% for the PC3 line under 21% and 1.5% oxygen, respectively, and 73.7 ± 5.0% and 65.1 ± 3.4% for the MCF-7 line under 21% and 1.5% oxygen, respectively. Surprisingly, these results suggest that BrdU is less cytotoxic to PC3 cells under hypoxic conditions than under normoxic conditions. In contrast, the sensitivity of the MCF-7 cell line to the studied derivative is slightly higher under hypoxic conditions, which might be connected to the fact that MCF-7 cells, contrary to PC3 cells, possess wild-type p53.

Furthermore, the shapes of the viability curves suggest that in the case of the PC3 cells, in the first 24 h, incubation with BrdU resulted in the inhibition of proliferation, which was characteristic for both oxygen variants. In the case of the MCF-7 cell line, the number of cells increased almost exponentially (Figure S3 in Supplementary Materials).

This experiment confirmed the low/acceptable cytotoxicity of BrdU in both cell lines. The results show that in the case of PC3, sensitivity to BrdU under hypoxic conditions, which are characteristic of solid tumors, was lower than under normoxic conditions. Moreover, the obtained results for the 48 h incubation with BrdU allowed for the selection of the radiosensitizer concentrations used in the next experiments, e.g., for the DSBs formation analysis. The selected concentrations were 1 µM and 10 µM. In the PC3 line incubated for 48 h with 1 µM BrdU, the viability was equal to 100.8 ± 2.9% (normoxic conditions) and to 98.6 ± 1.3% (hypoxic conditions). For a 10 µM concentration of BrdU, these values were equal to 99.17 ± 2.6% and 98.15 ± 1.6%, respectively. For the MCF-7 cells treated with the tested compound, the viability was reduced to 88.8 ± 5.6% (normoxic condition) and to 90.1 ± 1.6% (hypoxic condition). For the higher concentration, the viability values were equal to 79.0 ± 2.4% and 81.00 ± 3.2%, respectively.

### 2.4. Clonogenic Survival

**Figure 3:**
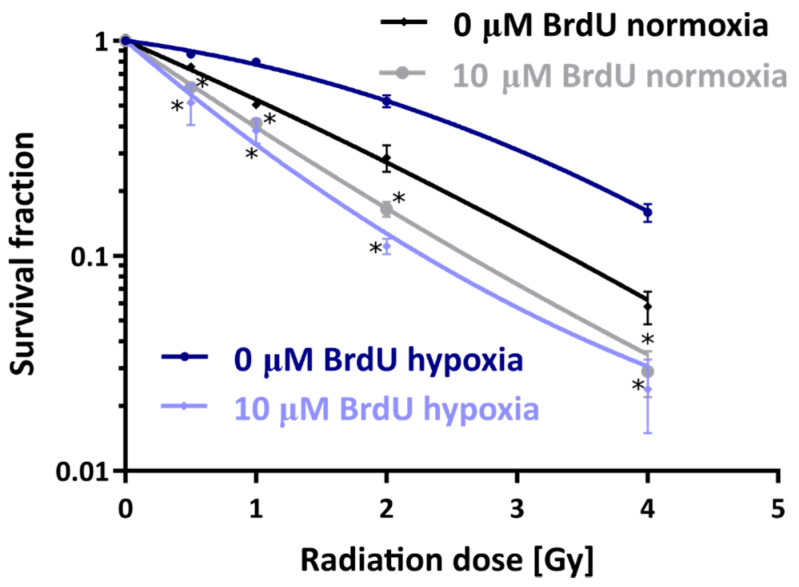
Dose–response curves for PC3 prostate cancer cells not treated or treated with BrdU (10 µM) under normoxia and hypoxia. Experiments were performed at least in duplicate, and the results are expressed as mean ± standard deviation. * Significant difference compared with the untreated variants, *p* < 0.05.

To study the influence of the oxygenation level on in vitro radiosensitization by BrdU, PC3 cells were incubated with the tested analog under hypoxic or normoxic conditions and subsequently irradiated. Figure 3 shows the survival fractions of cells irradiated with different doses under normoxia or hypoxia. As expected, in the absence of BrdU, hypoxia reduced the radiosensitivity of PC3 cells in comparison to normoxic conditions. The oxygen enhancement ratio (OER), representing the radiosensitizing effect of oxygen for a survival fraction (SF) equal to 0.5, was 1.92 (0 µM BrdU hypoxia vs. 0 µM BrdU normoxia). In cells pretreated with BrdU, the radiation response of hypoxic cells was enhanced to a much greater extent in comparison to normoxic cells. The sensitizer enhancement ratio (SER), representing the radiosensitizing properties of BrdU, was equal to 3.47 (0 µM BrdU hypoxia vs. 10 µM BrdU hypoxia) under hypoxia and 1.47 under normoxia (0 µM BrdU normoxia vs. 10 µM BrdU normoxia). Considering the values of SF, it can be observed that for pretreatment with the studied analog at a concentration of 10 µM, the survival of PC3 cells irradiated with 0.5 Gy was reduced from 75.8% to 60.7% under normoxia and from 86.7% to 51.6% under hypoxia. With a higher dose of 2 Gy after incubation with BrdU, SF was reduced from 28.6% to 16.5% under normoxia and from 52.3% to 11.1% under hypoxia. In order to compare the survival curves for cells treated with BrdU in normoxic and hypoxic conditions, the doses reducing the SF to 0.5 for both curves were estimated. For the curve corresponding to normoxic BrdU-treated cells, this parameter is equal to 0.742 Gy, and in the case of the curve corresponding to hypoxic cells treated with BrdU, this dose is equal to 0.606 Gy. Furthermore, based on survival curves, parameters for cellular radiosensitivity, such as α (coefficient for linear killing) and β (coefficient for quadratic killing) values, were calculated by fitting using the linear–quadratic model (SF = exp(−1 × (αD + βD^2^), where SF is the survival fraction and D is the dose). Average α and β values were as follows: 0.188 and 0.067 (untreated cells under hypoxia), 1.192 and −0.08 (cells treated with 10 µM BrdU under hypoxia), 0.609 and 0.021 (cells untreated under normoxia) and 0.955 and −0.029 (cells treated with 10 µM BrdU under normoxia). Since from a radiobiological point of view, negative values of the β parameter are not realistic [31], the fitting for curves corresponding to the survival of BrdU-treated cells was performed with β set to zero (see Figure S4). This procedure resulted in a slight change in α values: 1.101 (cells treated with 10 µM BrdU under hypoxia) and 0.919 (cells treated with 10 µM BrdU under normoxia). Regardless of the fitting procedure used, one should note that radiosensitization by BrdU is mainly due to an increase in the α linear parameter, which indicates an increase in the number of lethal events directly caused by BrdU. This observation remains in agreement with previous research [32], which suggested that an important mechanism of radiosensitization in cells substituted with halogenated pyrimidines involves an increase in effective DNA DSBs, and with the results of our studies on DSB formation discussed in Section 2.5. It should also be noted that the α value was enhanced by a factor of 6.3 (the ratio of α for the curve corresponding to cells treated with BrdU and untreated ones) under hypoxia and only 1.5 under normoxia. In general, the comparison of the survival curves (Figure 3) clearly shows that the oxygen level dramatically changes the radiosensitivity of the cancer cells pretreated with BrdU. The analog curves for the MCF-7 cell lines were not measured since, for the concentration of radiosensitizer suitable for PC3 (10 µM), BrdU was determined to be too toxic for reliable performance of the clonogenic test.

### 2.5. Double-Strand Break Formation

**Figure 4:**
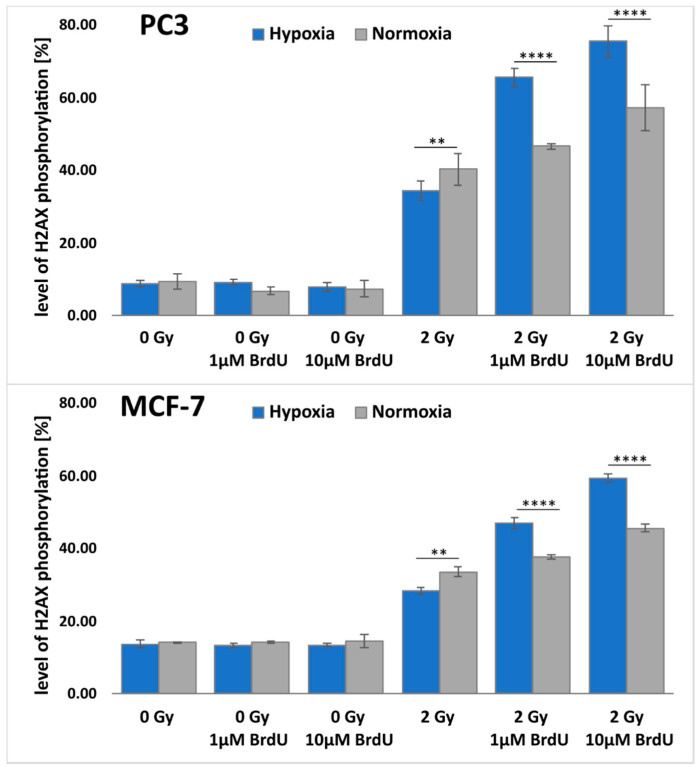
Flow cytometric analysis of H2A.X phosphorylation. γH2A.X was measured 1 h after irradiation. Results are shown as mean ± standard deviation of at least three independent experiments. The statistical significance was determined by one-way ANOVA followed by Tukey’s multiple comparison test: **** *p* < 0.0001, ** *p* < 0.01.

DSBs play a crucial role in radiation-induced cell killing. DNA damage resulting from BrdU and IR treatments was investigated by measuring the level of γH2A.X by flow cytometry (Figure 4 and Figure S5 in Supplementary Materials). The experiments were conducted on breast and prostate cancer cell lines, and the results were compared both in hypoxic and normoxic conditions. As expected, the number of DSBs (i.e., γH2A.X-positive cells) was significantly lower under hypoxic conditions compared to the normoxic ones for untreated breast and prostate cancer cells. With increasing BrdU concentration, the number of DSBs for irradiated cells increased. Moreover, in hypoxic cells pretreated with BrdU, the number of DSBs was larger than in the pretreated normoxic cells. For example, after incubation of normoxic PC3 cells with 10 µM BrdU and irradiation with a dose of 2 Gy, the level of γH2A.X increased from 40.44% (for the untreated control) to 57.45%. In hypoxic PC3 cells, treatment with 10 µM BrdU caused an increase in γH2A.X-positive cells from 34.49% (for the untreated and irradiated control) to 75.61%. In the case of the MCF-7 cell line, a similar relationship was observed. The results of cytometric analysis of H2A.X phosphorylation confirm that BrdU-mediated radiosensitization in vitro is dependent on the oxygenation level and is significantly greater under hypoxic conditions.

### 2.6. DNA Damage Signaling

Phosphorylation of histone variant H2A.X at Ser-139 (modification termed γH2A.X) is primarily mediated by ataxia-telangiectasia mutated (ATM) kinase in response to DSBs and is crucial for further signaling to proteins engaged in DNA repair [33,34]. ATM is activated by autophosphorylation at Ser-1981 [35]. Among its other important substrates is checkpoint kinase 2 (Chk2), which, upon phosphorylation at Thr-68, becomes activated and inhibits cell cycle progression [36], and p53, which, when phosphorylated at Ser-15, is stabilized and may act as a transcription factor that further enhances the cell cycle block and DNA repair or induces cell death [37,38].

**Figure 5:**
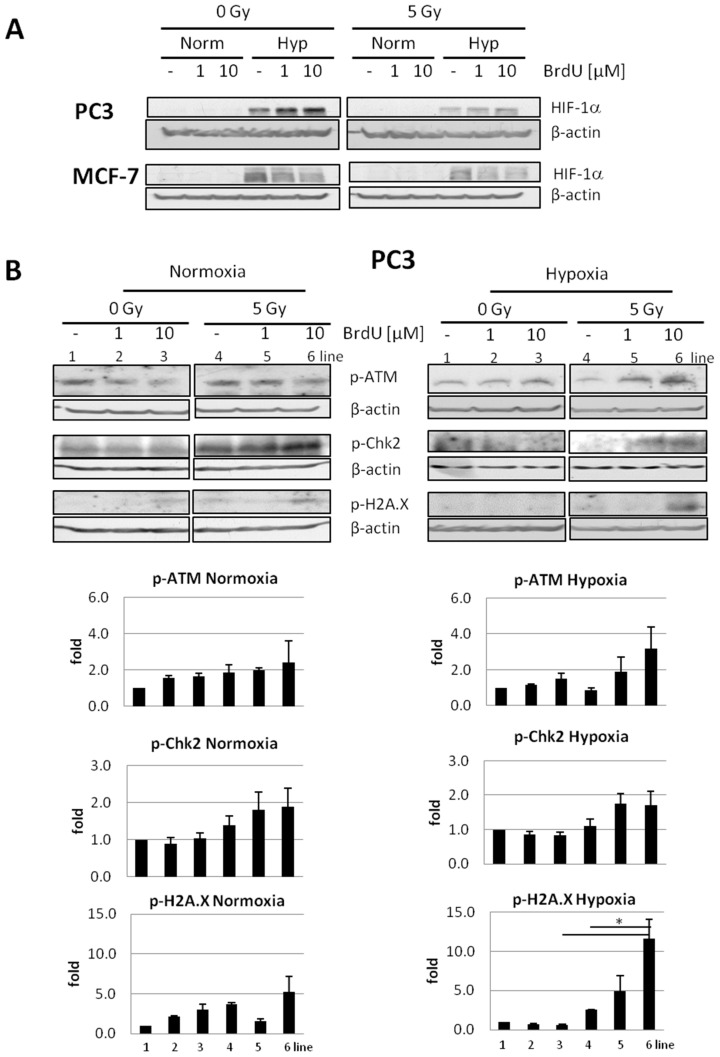

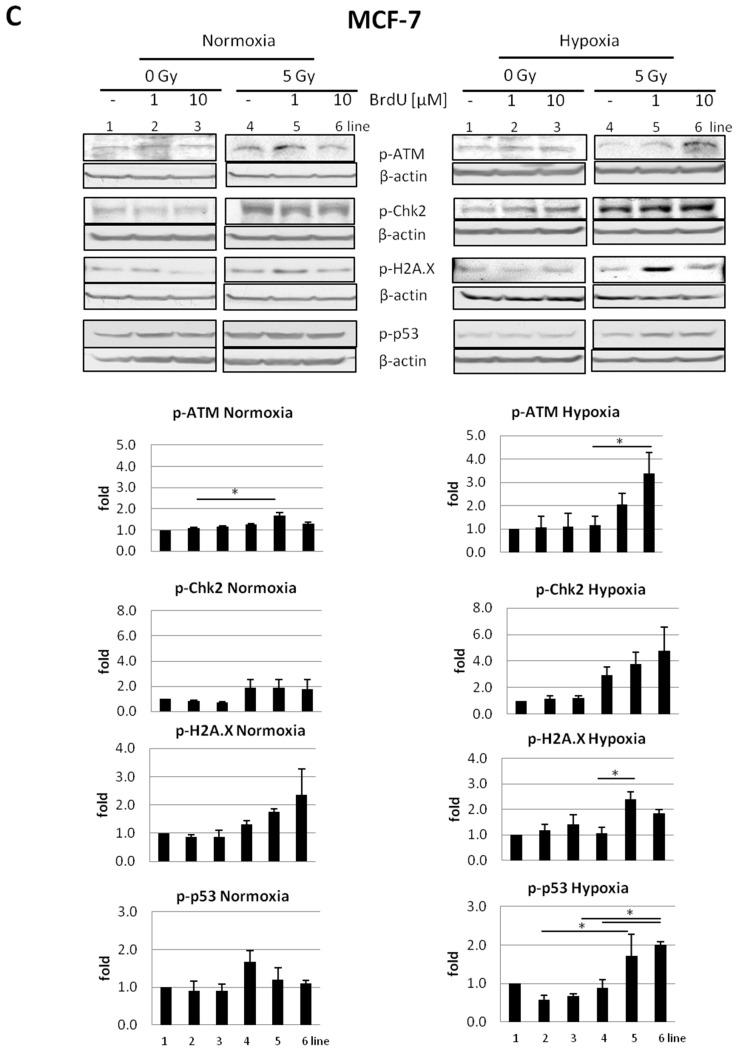
Effect of treatment with BrdU and/or exposure to ionizing radiation of cells in normoxic or hypoxic conditions. (**A**) Investigation of HIF-1α hypoxia marker in MCF-7 and PC3 cells untreated/treated (1 or 10 µM) with BrdU and unexposed/exposed to ionizing radiations (5 Gy) under hypoxia and normoxia conditions. Status of p-ATM, p-Chk2, p-H2A.X and p-p53 in (**B**) PC3 and (**C**) MCF-7 cells untreated/treated (1 or 10 µM) with BrdU and unexposed/exposed to ionizing radiations (5 Gy) under hypoxia and normoxia conditions. The blots were stripped and reprobed with anti-β-actin antibodies to normalize for differences in protein loading. Densitometric data, corrected for loading control, are graphed below (mean ± SE; *n* = 2–3). The statistical significance of differences was determined by one-way ANOVA followed by Sidak’s multiple comparison test, and * *p* < 0.05.

The status of these proteins in cells exposed to BrdU and/or ionizing radiation in normoxic or hypoxic conditions was investigated. HIF-1α was used as a marker of hypoxia, and as shown in Figure 5A, this protein was stabilized in cells cultured under 1.5% oxygen. In both cell lines, radiation (5 Gy) alone enhanced (although not at a statistically significant level) the phosphorylation of ATM and its substrates, Chk2 and H2A.X, to a higher level under normoxia than under hypoxia (Figure 5B,C). For instance, irradiation of PC3 cells elevated p-ATM by c.a. 2-fold under normoxia with no effect under hypoxia, p-Chk2 by 1.5-fold under normoxia and 1.1-fold under hypoxia, and p-H2A.X by 3.7-fold under normoxia compared to 2.6-fold under hypoxia (Figure 5B, lines 1 and 4 of respective blots and graphs). However, if cells were pretreated with BrdU, the phosphorylation of checkpoint proteins in irradiated hypoxic cells was comparable to or even higher than that in their normoxic counterparts (Figure 5B,C). For instance, in PC3 cells exposed to 10 μM BrdU compared to untreated controls, p-ATM was elevated by 2.4-fold and 3.2-fold; p-Chk2 was elevated by 1.8- and 1.7-fold; and p-H2A.X was elevated by 5.2- and 11.6-fold under normoxia and hypoxia, respectively (Figure 5B, line 6 in respective blots and graphs). In MCF-7 cells, which possess wt p53, phosphorylation of p53 at Ser-15 was also higher in irradiated cells cultured in normoxic compared to hypoxic conditions (1.7- vs. 0.9-fold), unless cells were labeled with BrdU. In this case, phosphorylation of p53 was higher in irradiated hypoxic (differences reached statistical significance) than normoxic cells compared to the respective cells not treated with IR (Figure 5C, lines 4–6 of p-p53 blots and graphs). These results indicate that under hypoxia, DNA damage signaling is indeed impaired, which is revealed by the lower activation level of the main checkpoint kinases, ATM and Chk2, in irradiated hypoxic cells compared to irradiated normoxic cells. Importantly, BrdU incorporated into DNA sensitizes hypoxic cells to IR and leads to the efficient activation of ATM, Chk-2 and p53 (in MCF-7 cells), which may result in proliferation inhibition or cell death with comparable efficiency to that observed in normoxic cells (Figure 3, for comparison).

## 3. Materials and Methods

### 3.1. Radiolysis

Radiolysis of BrdU solution at a concentration of 10^−4^ M containing 0.03 M *tert*-BuOH as a scavenger of ^●^OH radicals and phosphate buffer (10 mM, pH = 7.0) was carried out in a Cellrad X-ray cabinet (Faxitron X-ray Corporation, Tucson, AZ, USA). The irradiated samples were exposed to 140 Gy (4.14 Gy∙min^−1^, 130.0 kV, 5.0 mA, filter: 0.5 mm aluminum). One part of the sample was deoxygenated by purging with argon for 3 min. A reversed-phase HPLC method was employed for analysis of the irradiated and non-irradiated samples of BrdU with the use of a Dionex UltiMate 3000 system with Diode Array Detector (Dionex Corporation, Sunnyvale, CA, USA). The effluents were monitored at 260 nm. Separation by HPLC was achieved using a C18 column (Wakopak Handy ODS, 4.6 × 150 mm, 5 μm particle size and 100 Å pore size, Fujifilm Wako Chemicals, Osaka, Japan) with an isocratic elution of 0.1% HCOOH and 1% ACN. The studied samples were analyzed in triplicate. The chemicals used were purchased from Merck Millipore (Darmstadt, Germany).

### 3.2. Cell Culture

MCF-7 cells were grown in RPMI medium supplemented with 10% FBS and antibiotics at a concentration of 100 U·mL^−1^, and PC3 cells were cultured in F12K medium supplemented with 10% FBS and antibiotics at a concentration of 100 U·mL^−1^. The cell lines were obtained from ATCC (Manassas, VA, USA) and maintained at 37 °C in a humidified atmosphere with 5% CO_2_. All media and supplements were purchased from Gibco (Paisley, UK). The hypoxic group of cells was cultured in 1.5% O_2_ in an incubator with oxygen control (CB 60, Binder GmbH, Tuttlingen, Germany). The plates with cells were irradiated using a Cellrad X-ray cabinet. To achieve hypoxic conditions, irradiation and all manipulations were performed in a nitrogen atmosphere. 

### 3.3. Incorporation of BrdU into DNA

MCF-7 and PC3 cell lines were seeded into plates and incubated at 37 °C and 5% CO_2_ overnight. After that, the medium was replaced with fresh medium, and the cells were treated with BrdU at a concentration of 0 µM (control), 1 µM or 10 µM. Plates with cells were incubated with the compound in normoxic (21% of O_2_) or hypoxic conditions (1.5% of O_2_) for 48 h. After this time, the cells were removed from plates, and isolation of DNA was carried out according to the protocol provided by the manufacturer (GeneMATRIX Cell Culture DNA Purification Kit, EURX, Gdańsk, Poland). Then, purified DNA was enzymatically digested by the simultaneous action of nuclease P1, spleen phosphodiesterase, DNase I, snake venom phosphodiesterase (SVP) and bacterial alkaline phosphatase (BAP). Finally, the solution was chloroform-extracted to remove the enzymes, and the aqueous layer was lyophilized, resuspended in water and subjected to HPLC analysis. The isolated and digested DNA samples were analyzed with the use of the reversed-phase HPLC method on a Dionex UltiMate 3000 system with Diode Array Detector. The detection of effluents was carried out at 260 nm. Separations were performed using a Wakopak Handy ODS column (C18, 4.6 × 150 mm, 5 μm; 100 Å), 0.1% HCOOH in water as the mobile phase (isocratic elution) and a flow rate of 1 mL·min^−1^. The data were obtained from three experiments.

### 3.4. Viability Assay

To determine the cytotoxicity level of BrdU under hypoxic and normoxic conditions, the MTT assay was carried out. Cells from PC3 and MCF-7 lines were seeded into 96-well plates at a density of 4000 per well and incubated at 37 °C and 5% CO_2_ overnight. After that, the medium was replaced with fresh medium, and cells from both lines were treated with BrdU at 7 concentrations: 0 (for the control) 10^−4^, 10^−5^, 10^−6^, 10^−7^, 10^−8^ and 10^−9^ M. Next, plates with treated cells were incubated under normoxic (21% O_2_) or hypoxic (1.5% O_2_) conditions for 24, 48 and 72 h. After this time, 25 μL of an aqueous solution of MTT salt (4 mg mL^−1^) was added to each well and incubated for 4 h. Then, 200 µL of the medium with the MTT salt was removed, and DMSO was added. The absorbance was measured at 570 nm (660 nm was the reference wavelength) with an EnSpire microplate reader (PerkinElmer, Waltham, MA, USA). The vitality of the control was taken as 100%. The obtained results were analyzed with the GraphPad Prism 7 software (San Diego, CA, USA). The statistical significance was calculated using a one-way analysis of variance (ANOVA) followed by Dunnett’s multiple comparison test. The data were obtained from three experiments, and each treatment condition was assayed in triplicate. The differences were considered significant at *p* < 0.05.

### 3.5. Clonogenic Assay

Cells were plated at a density of 10^6^ cells per 60 mm dish and treated with BrdU at concentrations of 10 µM (PC3). After 48 h of treatment under normoxic (21% of O_2_) or hypoxic (1.5% of O_2_) conditions, the cells were irradiated with different doses (0–6 Gy). Delayed plating (after 6 h) was performed; cells were detached with Accutase and plated at a density of 800 cells per 100 mm dish. After 16 days, the resulting colonies were fixed with 6.0% (*v*/*v*) glutaraldehyde and 0.5% crystal violet. Stained colonies were counted manually, and colony size was assessed using an inverted fluorescence microscope (Olympus, IX73, Tokyo, Japan). The experiment was carried out in duplicate. Non-irradiated cultures were used as controls. The radiosensitizing effect of oxygen or BrdU is represented by the OER and SER, respectively (ID50 (−oxygen or treatment)/ID50 (+oxygen or treatment), where ID50 means the radiation dose causing 50% growth inhibition, determined by interpolation using the GraphPad Prism software). The obtained results were analyzed with the GraphPad Prism software using the linear–quadratic model. The statistical evaluation was calculated using a one-way analysis of variance (ANOVA) followed by Sidak’s multiple comparison test.

### 3.6. Histone H2A.X Phosphorylation Assay

Cells were treated with BrdU at a concentration of 0 µM (control), 1 µM or 10 µM and incubated (37 °C, 5% CO_2_) for 48 h under hypoxic (1.5% O_2_) or normoxic (21% O_2_) conditions. After this time, plates with cells were irradiated with 2 Gy and incubated for 1 h. Then, the cells were dissociated with Accutase solution, fixed, permeabilized and stained according to the manufacturer’s protocol (FlowCellect^TM^ Histone H2A.X Phosphorylation Assay Kit, Luminex, Austin, TX, USA) and then analyzed by flow cytometry (Guava easyCyte^TM^, Merck, Darmstadt, Germany). Untreated cultures were used as controls. The data were obtained from at least three experiments. The statistical significance of differences between the studied groups was determined by one-way ANOVA followed by Tukey’s multiple comparison test.

### 3.7. Western Blotting

Cells were treated with BrdU at a concentration of 0 µM (control), 1 µM or 10 µM and incubated (37 °C, 5% CO_2_) for 48 h under hypoxic (1.5% O_2_) or normoxic (21% O_2_) conditions. After this time, plates with cells were irradiated with 5 Gy and incubated for 1 h. Cells were collected, washed with ice-cold phosphate-buffered saline (PBS) and lysed in a solution containing 50 mM Tris, 1% Triton X-100, 150 mM NaCl, 0.5 mM EDTA and protease and phosphatase inhibitor cocktails (Roche Diagnostics Poland, Warsaw, Poland). The lysates were cleared by centrifugation at 15,500× *g* at 4 ℃ for 20 min. Proteins were resolved on 4–12% Bis-Tris gels (Thermo Fisher Scientific, Waltham MA, USA) by SDS–polyacrylamide gel electrophoresis (SDS-PAGE) with MES Running Buffer (Thermo Fisher Scientific, Waltham, MA, USA) and semi-dry transferred onto PVDF membrane (Thermo Fisher Scientific, Waltham, MA, USA). The membrane was blocked with 5% nonfat dry milk in PBS and incubated with the desired primary antibodies overnight at 4 °C. Antibodies against HIF-1α, p-ATM (Ser-1981), p-Chk2 (Thr-68), p-H2A.X (Ser-139) and p-p53 (Ser-15) were purchased from Cell Signaling Technology (Danvers, MA, USA). Next, the membrane was extensively washed with PBS and incubated with appropriate secondary antibodies for 1 h at room temperature. The immunoreactive bands were detected with enhanced chemiluminescence reagent (Clarity ECL Substrate; Biorad, Hercules, CA, USA) on X-ray films (Fujifilm, Tokyo, Japan). The blots were stripped and reprobed with anti-β-actin antibodies to normalize for differences in protein loading. The experiment was performed in 2 or 3 replicates.

## 4. Conclusions

Hypoxia is one of the main factors that reduce the effectiveness of radiotherapy. This fact was emphasized by Baumann and colleagues in their recent review regarding the radioresistance of neck and head cancers [39]. The authors suggested that the combination of hypoxia-sensitizing drugs with targeted radiation of hypoxic cells will become a gold standard of radiotherapy. Thus, the key role of a sensitizing drug in such a combined treatment prompted our studies on the effect of hypoxia on the cellular response of cells treated with BrdU, one of the best-known radiosensitizers, to IR. For comparison, all experiments were also performed under normoxic conditions.

One of the main properties of thymidine analogs as radiosensitizers is their effective electron-induced degradation. In this work, we demonstrated that the formation of uridine, as the main stable product of DEA to BrdU, was two-fold more effective under hypoxia, which reflects the fact that solvated electrons readily react with molecular oxygen to form peroxyl anion radicals that are much more inert than hydrated electrons. These data confirm that the pool of solvated electrons is higher at low oxygen levels. To perform their role, radiosensitizers with high electron affinity must be incorporated into DNA. As expected, the oxygen level did not affect the DNA labeling process. The incorporation of 5-bromo-2′-deoxyuridine depended on the time of treatment with radiosensitizer, its concentration and the cell line. On the other hand, hypoxia reduced the effectiveness of radiotherapy, which was confirmed by the clonogenic test. The results show that without BrdU, the cell response to IR (including activation of DNA damage signaling) was reduced under hypoxic conditions compared to normoxic cells. The situation was completely different in cells treated with BrdU, as hypoxia significantly increased the sensitivity of those cells to ionizing radiation. These results correlate with the formation of DSBs. For untreated cells, the DSB level after IR exposure was lower under hypoxic conditions. The opposite was observed for cells labeled with BrdU, where low oxygen concentration led to an increase in DSBs and DNA damage signaling.

Our studies confirm that BrdU is a hypoxic radiosensitizer; i.e., it works much better under hypoxia, which is one of the well-known obstacles for efficient radiotherapy.

## Figures and Tables

**Table 1:** Incorporation of BrdU [%] ^a^ applied at concentrations of 1 µM or 10 µM into genomic DNA. The experiment was performed in three replicates.

	Hypoxia		Normoxia	
	1 µM BrdU	10 µM BrdU	1 µM BrdU	10 µM BrdU
PC3, 24 h incubation	1.4 ± 0.5	11.2 ± 1.8	1.4 ± 0.3	12.1 ± 1.8
PC3, 48 h incubation	3.6 ± 0.1	23.2 ± 1.6	3.5 ± 0.1	24.2 ± 2.0
MCF-7, 24 h incubation	3.8 ± 0.5	16.6 ± 1.9	4.0 ± 0.5	16.1 ± 1.1
MCF-7, 48 h incubation	4.4 ± 0.4	24.3 ± 0.9	4.8 ± 0.8	23.7 ± 2.0

^a^ The percentage values were calculated based on HPLC peak areas corresponding to BrdU and dT (the ratios of BrdU area and the sum of BrdU and dT areas, corrected for the extinction coefficients of dT and BrdU, respectively) in the chromatograms of DNA samples digested to nucleosides.

## Data Availability

Research data are stored in an institutional repository and will be shared upon request to the corresponding author.

## Supplementary Materials

The following supporting information can be downloaded at: https://www.mdpi.com/article/10.3390/ijms23031429/s1.

## References

[1] De Ruysscher D., Niedermann G., Burnet N.G., Siva S., Lee A.W., Hegi-Johnson F. (2019). Radiotherapy Toxicity. Nat. Rev. Dis. Primers.

[2] Peters L.J., Withers H.R., Thames Jr H.D., Fletcher G.H. (1982). Keynote Address—The Problem: Tumor Radioresistance in Clinical Radiotherapy. Int. J. Radiat. Oncol. Biol. Phys..

[3] Rockwell S., Dobrucki I.T., Kim E.Y., Marrison S.T., Vu V.T. (2009). Hypoxia and Radiation Therapy: Past History, Ongoing Research, and Future Promise. Curr. Mol. Med..

[4] Gray L.H., Conger A., Ebert M., Hornsey S., Scott O.C.A. (1953). The Concentration of Oxygen Dissolved in Tissues at the Time of Irradiation as a Factor in Radiotherapy. Br. J. Radiol. Suppl..

[5] Evans S.M., Koch C.J. (2003). Prognostic Significance of Tumor Oxygenation in Humans. Cancer Lett..

[6] Wilson W.R., Hay M.P. (2011). Targeting Hypoxia in Cancer Therapy. Nat. Rev. Cancer.

[7] Hall E., Giaccia A. (2006). Radiobiology for the Radiologist.

[8] Bertout J., Patel S., Simon M. (2008). The Impact of O_2_ Availability on Human cancer. Nat. Rev. Cancer.

[9] Grimes D.R., Partridge M.A. (2015). Mechanistic Investigation of the Oxygen Fixation Hypothesis and Oxygen Enhancement Ratio. Biomed. Phys. Eng. Express..

[10] Wang H., Mu X., He H., Zhang X.D. (2018). Cancer Radiosensitizers. Trends Pharmacol. Sci..

[11] Gong L., Zhang Y., Liu C., Zhang M., Han S. (2021). Application of Radiosensitizers in Cancer Radiotherapy. Int. J. Nanomed..

[12] Fowler J.F., Adams G.E., Denekamp J. (1976). Radiosensitizers of Hypoxic Cells in Solid Tumours. Cancer Treat. Rev..

[13] Zdrowowicz M., Chomicz-Mańka L., Butowska K., Spisz P., Falkiewicz K., Czaja A., Rak J., Leszczynski J., Shukla M.K. (2021). DNA Damage Radiosensitizers Geared Towards Hydrated Electrons. Practical Aspects of Computational Chemistry.

[14] Boudaïffa B., Cloutier P., Hunting D., Huels M.A., Sanche L. (2000). Resonant Formation of DNA Strand Breaks by Low-Energy (3 to 20 eV) Electrons. Science.

[15] Scholes G., Hüttermann J., Köhnlein W., Teoule R. (1978). Effects of Ionizing Radiation on DNA. Physical, Chemical and Biological Aspects.

[16] Nabben F.J., Karman J.P., Loman H. (1982). Inactivation of Biologically Active DNA by Hydrated Electrons. Int. J. Radiat. Biol. Relat. Stud. Phys. Chem. Med..

[17] Wang W., Sevilla M.D. (1994). Protonation of Nucleobase Anions in Gamma-Irradiated DNA and Model Systems. Which DNA Base is the Ultimate Sink for the Electron?. Radiat. Res..

[18] Kohanoff J., McAllister M., Tribello G.A., Gu B. (2017). Interactions Between Low Energy Electrons and DNA: A Perspective from First-Principles Simulations. J. Phys. Condens. Matter.

[19] Rak J., Chomicz L., Wiczk J., Westphal K., Zdrowowicz M., Wityk P., Żyndul M., Makurat S., Golon Ł. (2015). Mechanisms of Damage to DNA Labeled with Electrophilic Nucleobases Induced by Ionizing or UV Radiation. J. Phys. Chem. B.

[20] Chomicz L., Zdrowowicz M., Kasprzykowski F., Rak J., Buonaugurio A., Wang Y., Bowen K.H. (2013). How to Find out Whether a 5-Substituted Uracil Could be a Potential DNA Radiosensitizer. J. Phys. Chem. Lett..

[21] Chomicz-Mańka L., Wityk P., Golon Ł., Zdrowowicz M., Wiczk J., Westphal K., Żyndul M., Makurat S., Rak J., Leszczynski J., Kaczmarek-Kedziera A., Puzyn T., Papadopoulos M.G., Shukla M.K. (2017). Consequences of Electron Attachment to Modified Nucleosides Incorporated into DNA. Handbook of Computational Chemistry.

[22] Lee L.-S., Cheng Y. (1976). Human Deoxythymidine Kinase. 2. Substrate Specificity and Kinetic Behaviors of the Cytoplasmic and Mitochondria Isozymes Derived from the Blast Cells of the Acute Myelocytic Leukemia. Biochemistry.

[23] Goz B. (1977). The Effects of Incorporation of 5-halogenated Deoxyuridines into the DNA of Eukaryotic Cells. Pharmacol. Rev..

[24] Erikson R.L., Szybalski W. (1963). Molecular radiobiology of human cell lines. V. Comparative radiosensitizing properties of 5-halodeoxycytidines and 5-halodeoxyuridines. Radiat. Res..

[25] Kinsella T.J., Dobson P.P., Mitchell J.B., Fornace A.J. (1987). Enhancement of X Ray Induced DNA Damage by Pre-treatment with Halogenated Pyrimidine Analogs. Int. J. Radiat. Oncol. Biol. Phys..

[26] Prados M.D., Scott C., Sandler H., Buckner J.C., Phillips T., Schultz C., Urtasun R., Davis R., Gulin P., Cascino T. (1999). A Phase 3 Randomized Study of Radiotherapy Plus Procarbazine, CCNU, and Vincristine (PCV) with or without BUdR for the Treatment of Anaplastic Astrocytoma: A Preliminary Report of RTOG 9404. Int. J. Radiat. Oncol. Biol. Phys..

[27] Groves M.D., Maor M.H., Myers C., Kyritsi A.P., Jaeckle K.A., Yung W.K.A., Sawaya R.E., Hess K., Bruner J.M., Peterson P. (1999). A Phase II Trial of High-Dose Bromodeoxyuridine with Accelerated Fractionation Radiotherapy Followed by Procarbazine, Lomustine and Vincristine for Glioblastoma Multiforme. Int. J. Radiat. Oncol. Biol. Phys..

[28] Spisz P., Zdrowowicz M., Makurat S., Kozak W., Skotnicki K., Bobrowski K., Rak J. (2019). Why Does the Type of Halogen Atom Matter for the Radiosensitizing Properties of 5-Halogen Substituted 4-Thio-2′-Deoxyuridines?. Molecules.

[29] Iliakis G., Kurtzman S., Pantelias G., Okayasu R. (1989). Mechanism of Radiosensitization by Halogenated Pyrimidines: Effect of BrdU on Radiation Induction of DNA and Chromosome Damage and its Correlation with Cell Killing. Radiat. Res..

[30] Dillehay L.E., Thompson L.H., Carrano A.V. (1984). DNA-Strand Breaks Associated with Halogenated Pyrimidine Incorporation. Mutat. Res..

[31] Van Leeuwen C.M., Oei A., Crezee J., Bel A., Franken N., Stalpers L., Kok H. (2018). The Alfa and Beta of Tumours: A Review of Parameters of the Linear-quadratic Model, Derived from Clinical Radiotherapy Studies. Radiat. Oncol..

[32] Franken N., Oei A., Kok H., Rodermond H., Sminia P., Crezee J., Stalpers L., Barendsen G. (2013). Cell Survival and Radiosensitisation: Modulation of the Linear and Quadratic Parameters of the LQ Model. Int. J. Oncol..

[33] Petrini J.H., Stracker T.H. (2003). The Cellular Response to DNA Double-Strand Breaks: Defining the Sensors and Mediators. Trends Cell Biol..

[34] Rogakou E.P., Pilch D.R., Orr A.H., Ivanova V.S., Bonner W.M. (1998). DNA Double-Stranded Breaks Induce Histone H2AX Phosphorylation on Serine. J. Biol. Chem..

[35] Bakkenist C.J., Kastan M.B. (2003). DNA Damage Activates ATM through Intermolecular Autophosphorylation and Dimer Dissociation. Nature.

[36] Bartek J., Falck J., Lukas J. (2001). CHK2 Kinase—A Busy Messenger. Nature Rev. Mol. Cell Biol..

[37] Banin S., Moyal L., Shieh S., Taya Y., Anderson C.W., Chessa L., Smorodinsky N.I., Prives C., Reiss Y., Shiloh Y. (1998). Enhanced Phosphorylation of p53 by ATM in Response to DNA Damage. Science.

[38] Siliciano J.D., Canman C.E., Taya Y., Sakaguchi K., Appella E., Kastan M.B. (1997). DNA Damage Induces Phosphorylation of the Amino Terminus of p53. Genes Dev..

[39] Baumann R., Depping R., Delaperriere M., Dunst J. (2016). Targeting Hypoxia to Overcome Radiation Resistance in Head & Neck Cancers: Real Challenge or Clinical Fairytale?. Expert Rev. Anticancer. Ther..

