# Supplementary material for: Influence of Hypoxia on Radiosensitization of Cancer Cells by 5-Bromo-2′-deoxyuridine
Source: Int J Mol Sci. 2022 Jan 27;23(3):1429. doi: 10.3390/ijms23031429 (PMC8836255; doi:10.3390/ijms23031429)
Supplement: Supplementary file 1 [file ijms-23-01429-s001.zip › ijms-1510798-supplementary.pdf]

Supplementary Materials

Influence of hypoxia on radiosensitization of cancer cells by 5-bromo-2′-deoxyuridine

Magdalena Zdrowowicz[1], Paulina Spisz[1], Aleksandra Hać[2], Anna Herman-Antosiewicz[2], Janusz Rak[1]*

[1]Laboratory of Biological Sensitizers, Faculty of Chemistry, University of Gdańsk, 80-308 Gdańsk, Poland;
[2]Department of Medical Biology and Genetics, Faculty of Biology, University of Gdansk, 80-308, Gdańsk, Poland;

* Correspondence: janusz.rak@ug.edu.pl (J.R.); Tel.: +48 58 523 51 18

Table of Content

Identification of radiolysis product (Figure S1)……………………….……………........S3

Incorporation of BrdU into genomic DNA - HPLC analyses (Figure S2)...………..……S4

Cytotoxicity (Figure S3)...……………………………………..………………………S9

Survival curves (Figure S4)……………………………………...…………….….….S10

Cytometric analysis of histone H2A.X phosphorylation (Figure S5)………………..….S11

Identification of radiolysis product

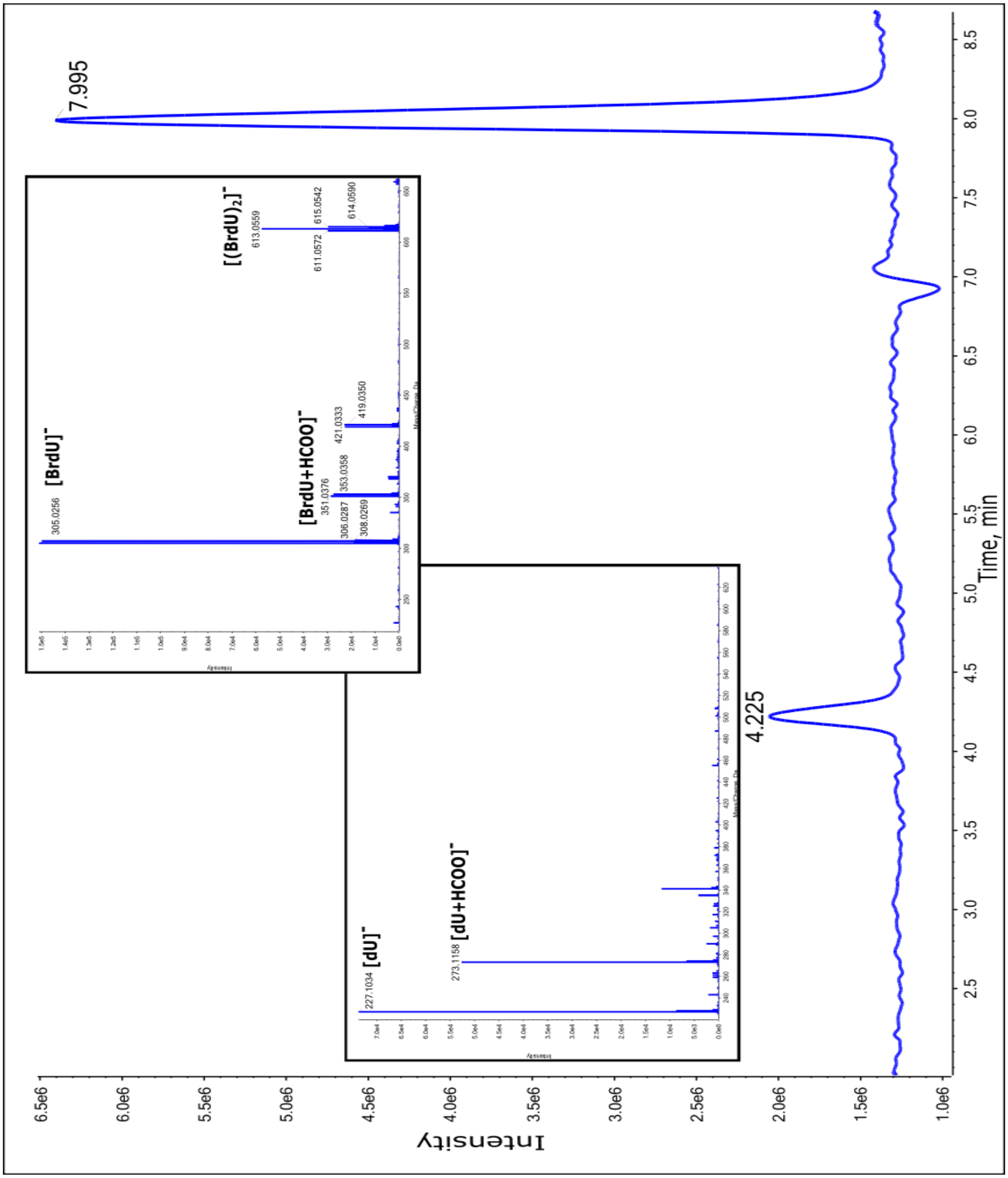

Figure S1. Identification of radiolysis product – LC-MS analysis. TIC (Total Ion Current) of solution after radiolysis (in negative ionization mode) and MS spectra of radiolysis products.

Conditions of chromatographic separations: Kinetex column (Phenomenex, 2.1 × 150mm, 2,6 µm, 100 Å), gradient elution with: phase A: 0.2% HCOOH and phase B: 80% ACN (0-5 min 0% B; 5-30 min from 0 to 100% B); flow rate 0.3 mL · min^{-1}. Conditions of MS analysis: the spray voltage -4.5 kV; source temperature 300˚C.

Incorporation of BrdU into genomic DNA - HPLC analyses

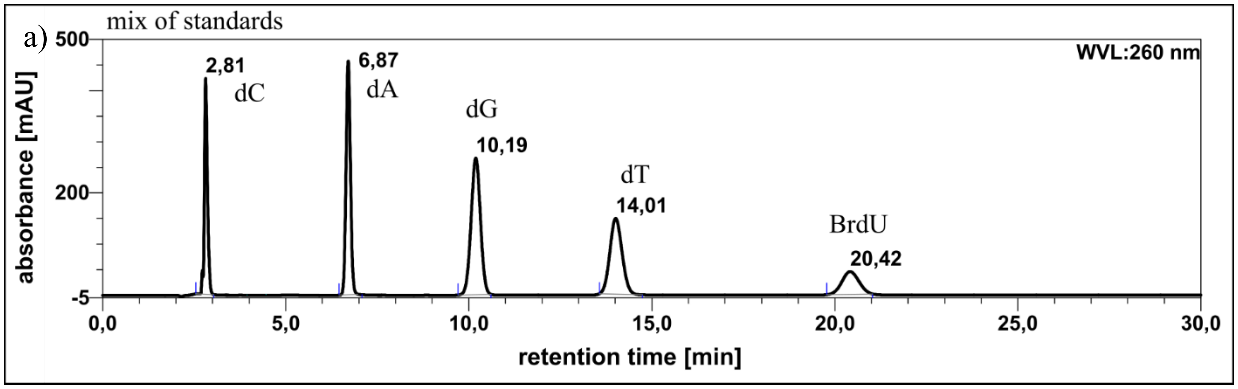

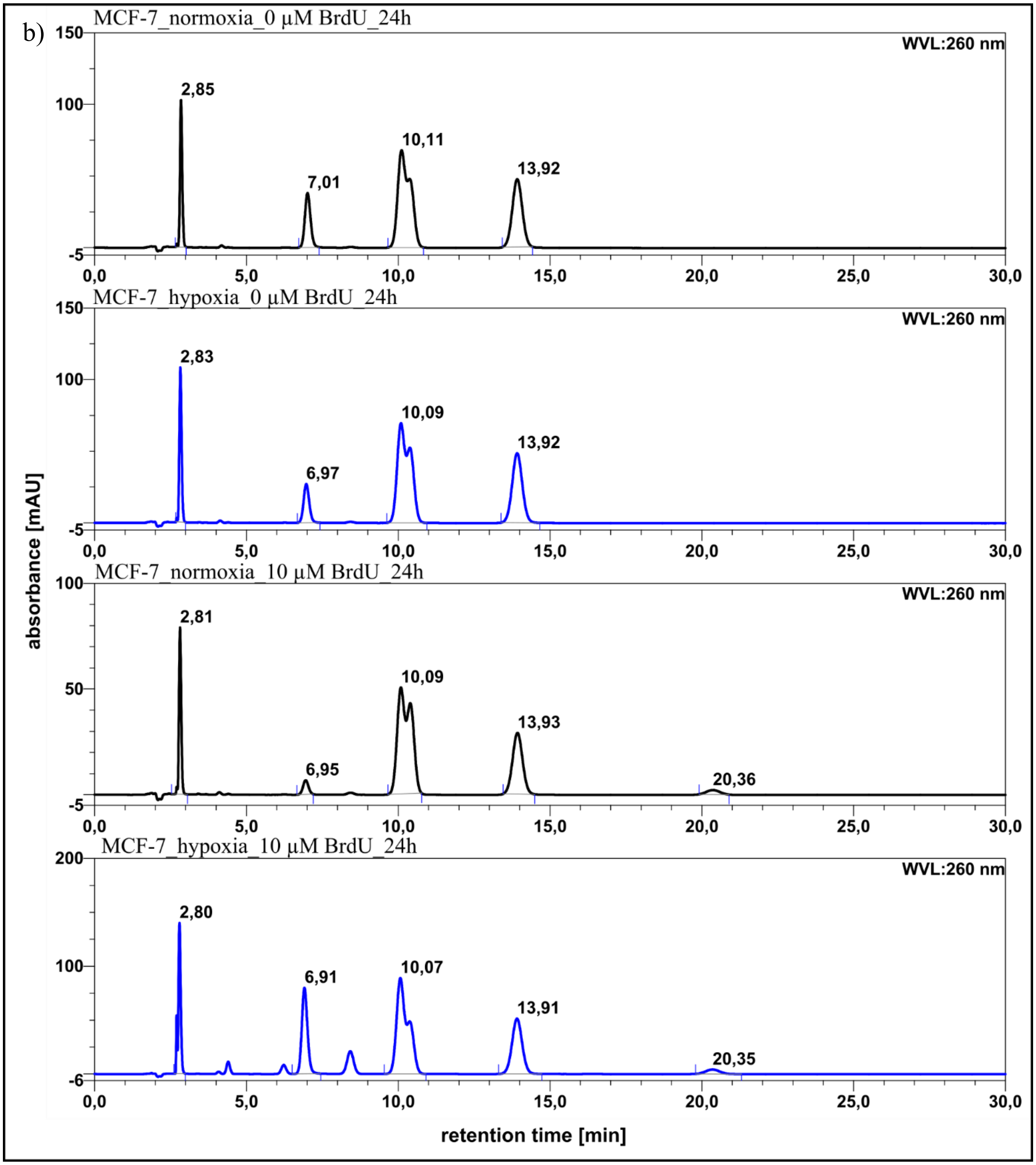
b)
MCF-7_normoxia_0 µM BrdU_24h
WVL:260 nm
2,85
7,01
10,11
13,92
MCF-7_hypoxia_0 µM BrdU_24h
WVL:260 nm
2,83
6,97
10,09
13,92
MCF-7_normoxia_10 µM BrdU_24h
WVL:260 nm
2,81
6,95
10,09
13,93
20,36
MCF-7_hypoxia_10 µM BrdU_24h
WVL:260 nm
2,80
6,91
10,07
13,91
20,35
absorbance [mAU]
retention time [min]

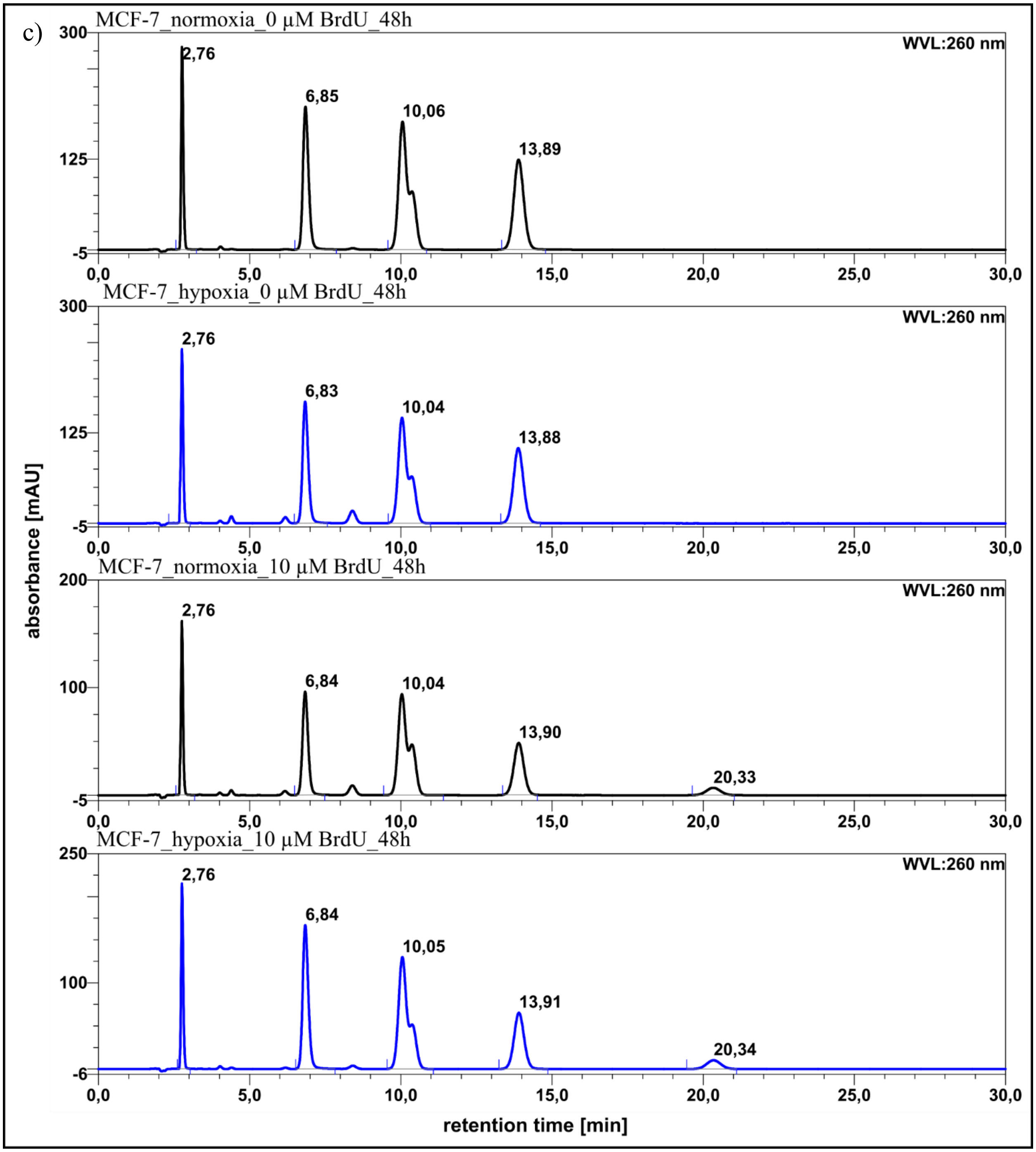
c)
MCF-7_normoxia_0 µM BrdU_48h
WVL:260 nm
2,76
6,85
10,06
13,89
MCF-7_hypoxia_0 µM BrdU_48h
WVL:260 nm
2,76
6,83
10,04
13,88
MCF-7_normoxia_10 µM BrdU_48h
WVL:260 nm
2,76
6,84
10,04
13,90
20,33
MCF-7_hypoxia_10 µM BrdU_48h
WVL:260 nm
2,76
6,84
10,05
13,91
20,34
absorbance [mAU]
retention time [min]

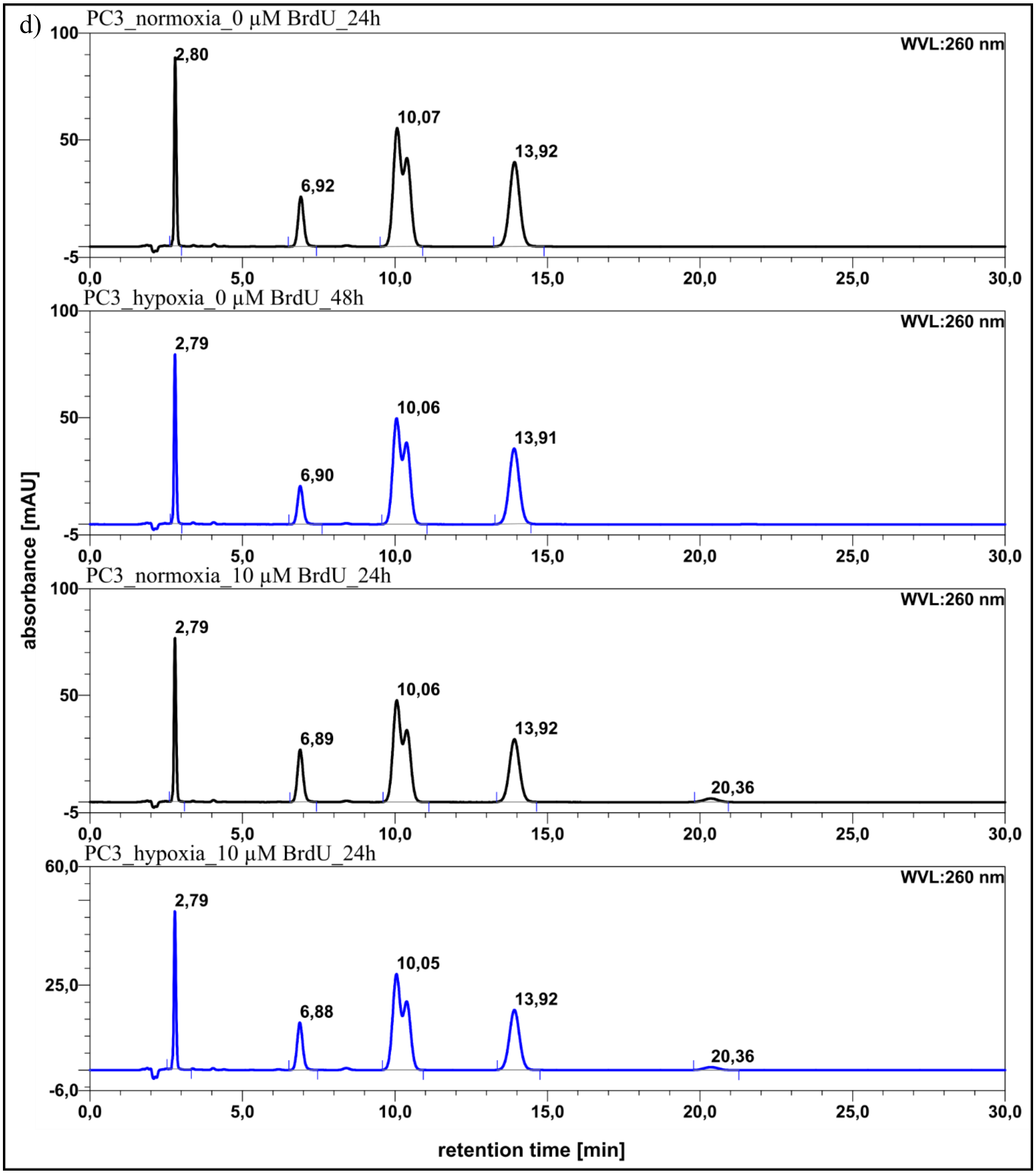
d)
PC3_normoxia_0 µM BrdU_24h
WVL:260 nm
2,80
6,92
10,07
13,92
PC3_hypoxia_0 µM BrdU_48h
WVL:260 nm
2,79
6,90
10,06
13,91
PC3_normoxia_10 µM BrdU_24h
WVL:260 nm
2,79
6,89
10,06
13,92
20,36
PC3_hypoxia_10 µM BrdU_24h
WVL:260 nm
2,79
6,88
10,05
13,92
20,36
absorbance [mAU]
retention time [min]

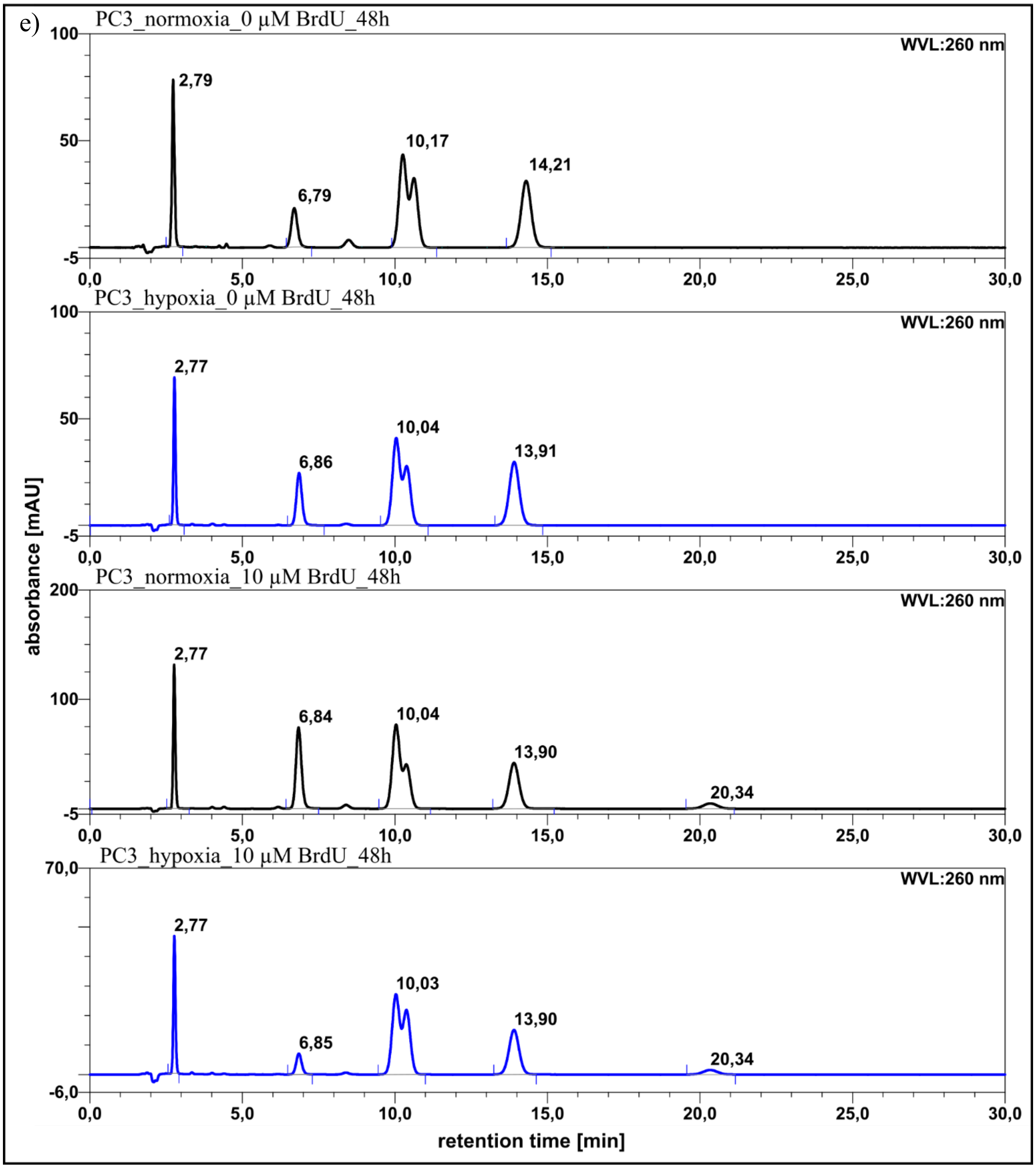

Figure S2. Incorporation of BrdU into genomic DNA - HPLC analysis. Exemplary HPLC traces for a) mixture of nucleosides – standards and enzymatic digested DNA sample isolated from: b,c) MCF-7 cells or d,e) PC3 cells. Double signal with retention time ~10 min corresponds to the 2'-deoxyguanosine and 2'-deoxyinosine resulting from enzymatic digestion (adenosine deaminase activity of commercial spleen phosphodiesterase).

Cytotoxicity

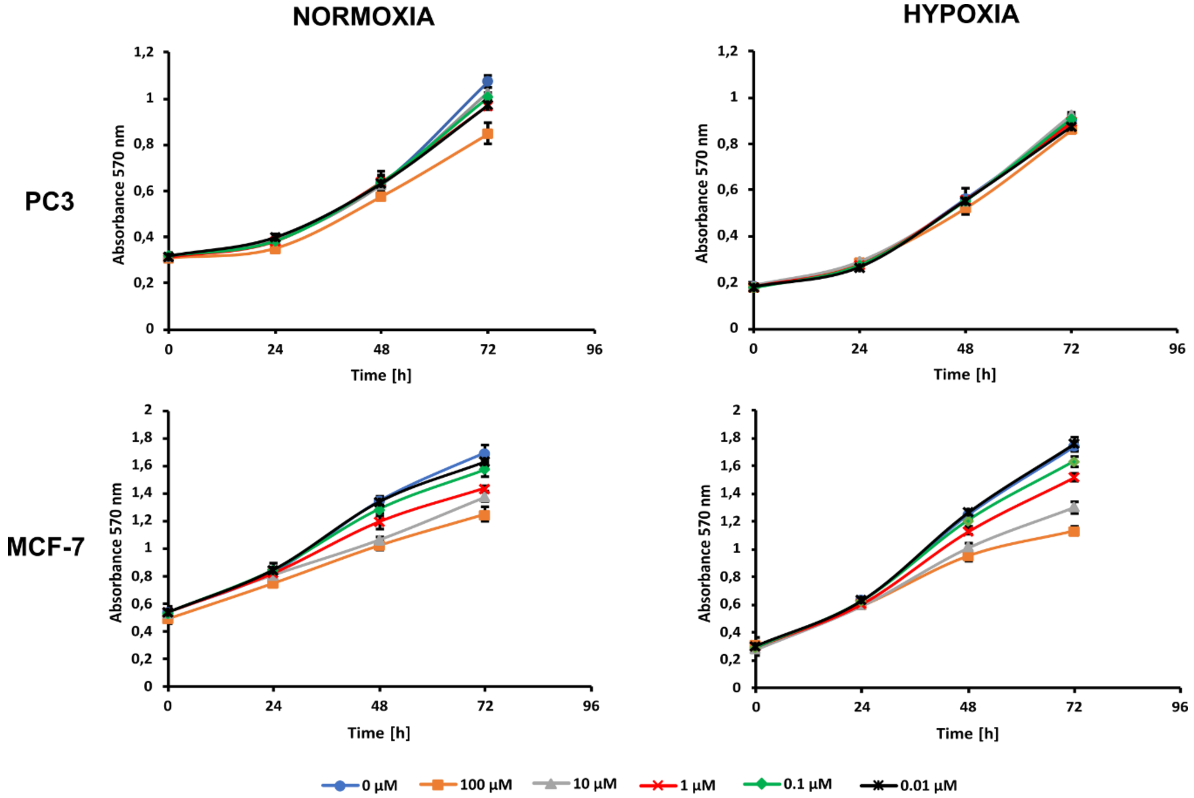

Figure S3. Viability of MCF-7 and PC3 cells measured using MTT assay exposed to different concentrations of BrdU under hypoxia or normoxia conditions. The absorbance at 570 nm is plotted as a function of time. Results are shown as mean ± standard deviation (SD) of three independent experiments performed in triplicate.

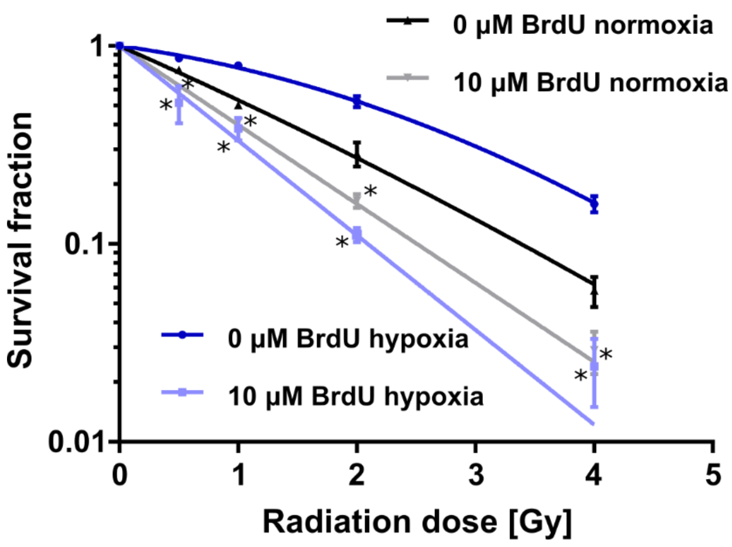

Figure S4. Dose response curves for PC3 prostate cancer cells non treated or treated with BrdU (10 μM) under normoxia and hypoxia. The fitting has been performed with β forced to zero for curves corresponding to survival of BrdU-treated cells.

Cytometric analysis of histone H2A.X phosphorylation

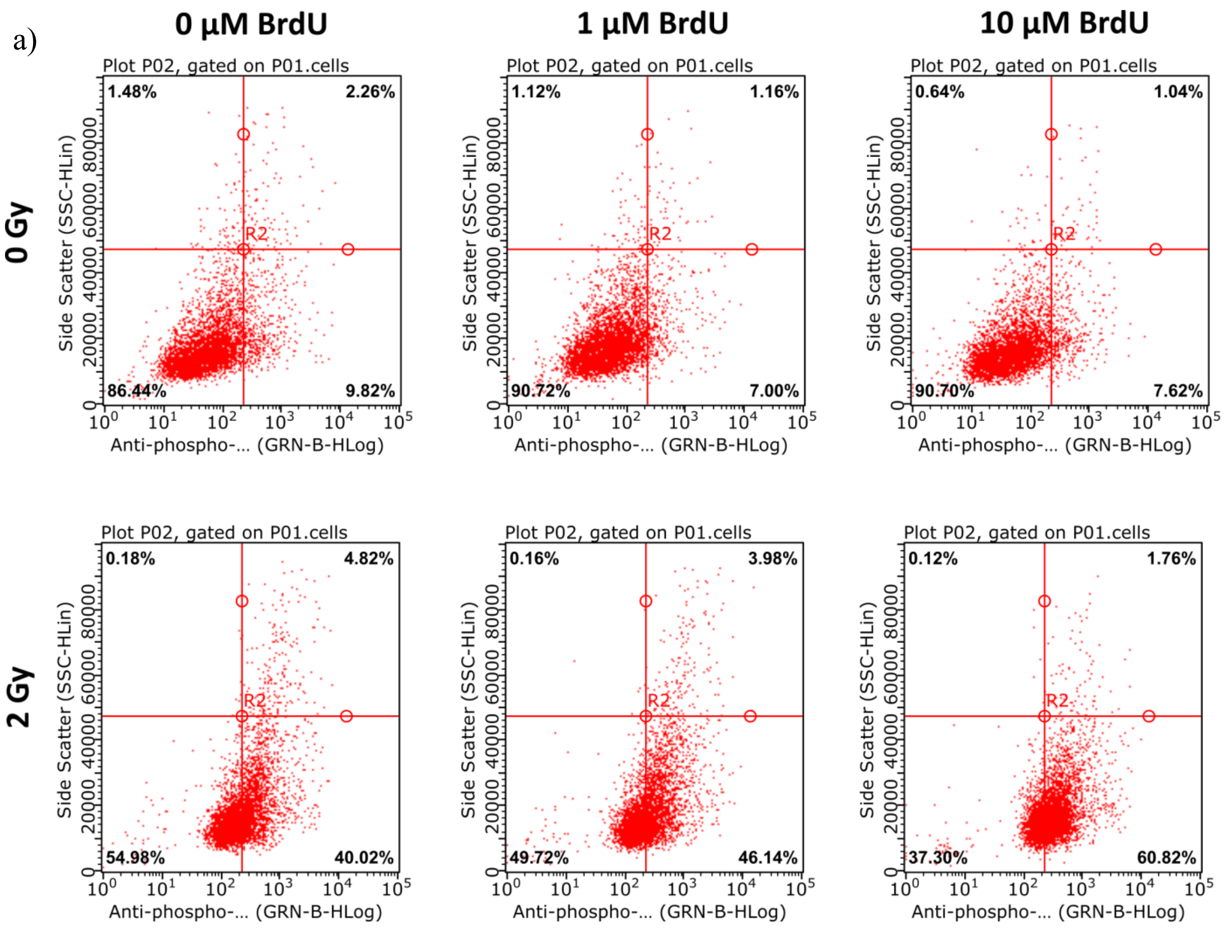

b)

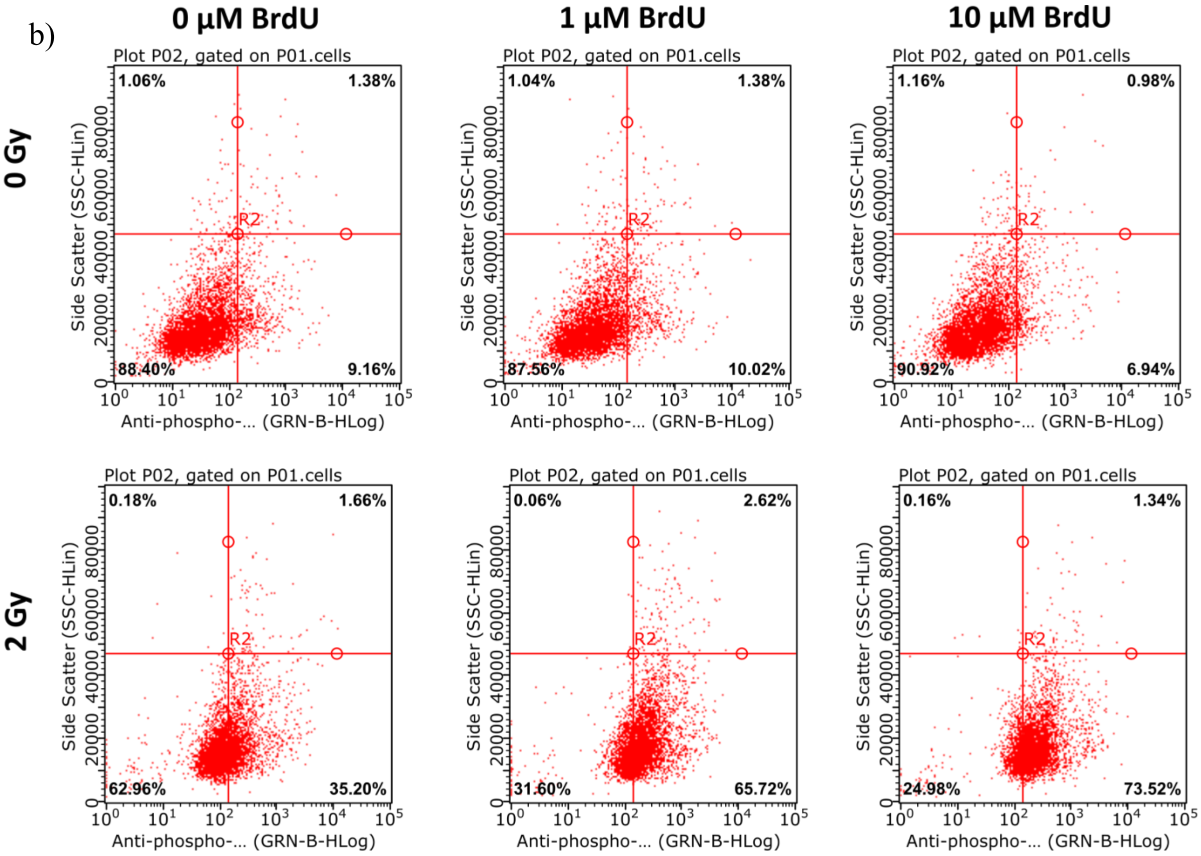

Figure S5. Exemplary cytometric analysis of histone H2A.X phosphorylation for a) normoxic PC3 cells, b) hypoxic PC3 cells.
